# Performance of age-adjusted D-dimer values for predicting DVT before the knee and hip arthroplasty

**DOI:** 10.1186/s13018-020-02172-w

**Published:** 2021-01-25

**Authors:** Jian Xiang Wu, Jiang Hui Qing, Yao Yao, Dong Yang Chen, Qing Jiang

**Affiliations:** 1 Department of Sports Medicine and Adult Reconstructive Surgery, Drum Tower Hospital, School of Nanjing Medical University, 321 Zhongshan Road, Nanjing, 210008 Jiangsu P.R. China; 2 State Key Laboratory of Pharmaceutical Biotechnology, Department of Sports Medicine and Adult Reconstructive Surgery, Nanjing Drum Tower Hospital, The Affiliated Hospital of Nanjing University Medical School, 321 Zhongshan Road, Nanjing, 210008 Jiangsu P.R. China

## Abstract

**Purpose:**

To compare the specificity and sensitivity of preoperative D-dimer and age-adjusted D-dimer value for predicting the incidence of the DVT preoperatively in total joint arthroplasty (TJA) patients.

**Methods:**

We enrolled 406 patients finally above 50 years old. Everyone had done ultrasonography bedside, and D-dimer concentrations were collected before surgery. The D-dimer and age-adjusted D-dimer cut-off was calculated by multiple logistic regression and receiver operating curve (ROC) analyses.

**Results:**

A total of 39 patients had found asymptomatic deep vein thrombosis (DVT) by ultrasonography. The age (odds ratio [OR] 1.067; *p* = 0.003) and D-dimer (OR 1.331; *p* = 0.025) were related to the existence of DVT. For conventional D-dimer and age-adjusted D-dimer value, the area under the curves (AUCs) were 0.685 (0.499–0.696) and 0.795 (0.611–0.881), respectively.

**Conclusion:**

Compared to traditional D-dimer, age-adjusted D-dimer showed better performance in screening DVT, which was useful clinically.

**Supplementary Information:**

The online version contains supplementary material available at 10.1186/s13018-020-02172-w.

## Introduction

As is known to all, the total hip arthroplasty (THA) and total knee arthroplasty (TKA) are the effective methods to treat osteoarthritis, but it also can bring some complications, such as infection, pain, and deep vein thrombosis (DVT), which was the most common complication. The incidence of DVT is about 11.9% in THA and 20.8% in TKA after surgery [1]. DVT can lead to Pulmonary thromboembolism (PTE), which is the most serious complication after arthrosurgery. There are many researches that focus on the incidence of DVT postoperatively; few studies about preoperative DVT were reported [2]. In clinical work, the removal of thrombus preoperatively may need the help of imaging examination such as ultrasound and contrast radiography. However, there are inadequacies in all the above examinations, such as time-consuming, expensive, and labor-consuming, so they are not suitable for large-scale use before operation. If patients have asymptomatic DVT preoperatively, it might cause severe consequence. Thus, a valuable method in detection of DVT or PTE before surgery is necessary.

In clinical work, the prompt diagnosis for thrombosis is the D-dimer test, which can give references to the surgeon [3]. D-dimer is produced by fibrin degradation [4]. The monitoring value of D-dimer is identified according to different methods. The cut-off value of D-dimer is 0.5 μg/ml by latex agglutination turbidimetry (LATEX) in our hospital; however, in the elderly patients, the D-dimer concentrations are higher than the upper limit of normal age [5]. Thus, the value of D-dimer is discounted in the elderly patients [6]. Moreover, the pregnancy, infection, inflammation, and cancer also can lead to high D-dimer value. As a result, the only use of D-dimer to predict DVT is not appropriate.

Some studies showed that the use of age-adjusted D-dimer cut-off in the diagnostic strategy for the specificity and sensitivity of deep vein thrombosis is more valuable over 50 years old [7, 8]. Nybo and Hvas argued that the use of an age-adjusted D-dimer in patients above 50 years of age for ruling out DVT seems as safe as using a standard D-dimer cut-off [9]. Schouten et al. also described that the application of age-adjusted cut-off values to the D-dimer value (0.01 × age [years]) considerably elevates specificity without altering sensitivity in patients aged > 50 years with suspected PTE [10].

Therefore, the use of D-dimer concentrations for predicting deep vein thrombosis is controversial [11]. However, the application of age-adjusted cut-off values to the D-dimer value is relatively rare in orthopedics. So, it is valuable for us to research. The method of this study is to compare the specificity and sensitivity of preoperative D-dimer and age-adjusted D-dimer value for predicting the incidence of the DVT preoperatively. Our aim is to explore the feasibility and effectiveness of this method in the field of joint replacement.

## Materials and methods

We enrolled 834 patients, whose age was over 50 years old, who underwent THA or TKA at Affiliate Drum Tower Hospital, Medical School of Nanjing University from September 1, 2015, to April 1, 2017. All patients signed the informed consent. The patients who underwent the pregnancy, infection, and cancer were excluded. In addition, patients were excluded if they had a femoral neck fracture, whose concentration of D-dimer was above normal level. Also, patients with cardiovascular disease and a previous history of thromboembolism were excluded. We removed the patients who had taken anticoagulant or antiplatelet agents previously. In the end, we included 406 patients in our study. All the patients drew blood for collecting the plasma D-dimer level when they were admitted to hospital. The cut-off value of D-dimer was 0.5 μg/ml by latex agglutination turbidimetry (LATEX). We divided patients aged > 50 into 10-year age groups. Renee A Douma made three large cohorts to find the age-adjusted D-dimer value. They plotted the D-dimer cut-off level against age group and performed linear regression analysis to obtain the regression coefficient. They finally proved the multiplication factor for age in the new age-adjusted cut-off value was 0.01. Thus, the age-adjusted D-dimer value was calculated as age (years) × 0.01 μg/mL. The plasma D-dimer level, diagnosis, age, sex, and body mass index (BMI) of the patients were recorded preoperatively. All the patients did not take anticoagulant or antiplatelet agents. The surgeries were done by four skilled orthopedic surgeons. The patients accepted the same program of rehabilitation and postoperative management. We observed vein thrombosis 1 day preoperatively. The skilled radiologists performed thrombosis examinations for all patients. The double lower limbs were observed with bedside ultrasound machine with two mode (B-mode and color Doppler ultrasound examination) for the common femoral, popliteal, superficial, and calf veins bilaterally. DVT was diagnosed if (1) the vein could not be compressed and (2) there were abnormal vascular signals in Doppler color ultrasound. Plasma D-dimer normal value was defined as 0.5 μg/ml. We wanted to analyze the age-adjusted D-dimer cut-off to predict the DVT event. At the same time, we also analyzed the risk factors for preoperative deep venous thrombosis. We predicted that the age-adjusted D-dimer was a more valuable index for monitoring the DVT before the TKA and THA.

All statistical analyses were from SPSS (version 25; SPSS Inc., Chicago, IL, USA). The qualitative data such as the number of females or males was analyzed by Fisher’s exact test. The quantitative data such as body height, body weight, age, body mass index, and D-dimer value was analyzed by unpaired Student’s *t* test. Multiple logistic regression analysis was used to discover the following risk factors related to the presence of DVT: body height, body weight, body mass index (BMI), age, sex, and D-dimer value. Receiver operating characteristic (ROC) curves were produced for age-adjusted D-dimer value and conventional D-dimer value. Also, the area under the curve (AUC) was calculated by ROC curves. In addition, we used the Youden index [12] to determine the cut-off value. We considered *p* < 0.05 to be statistically significant.

## Results

In the end, we included 406 patients. There were 118 males and 288 females. The average BMI of all patients was 25.3 ± 3.9 kg/m^2^. The mean age was 65.4 ± 8.4 years old. The patients’ detailed preoperative basic data and related blood tests are shown in Table 1.

**Table 1:** Patients’ characteristics

	Total patients	Patients with DVT	Patients without DVT	*p* value
Patient (*n*)	406	39	367	-
Sex (male/female)	118/288	9/30	109/258	0.386
Age (years)	65.4 ± 8.4	69.8 ± 6.9	65.0 ± 8.5	< 0.001
Body weight (kg)	66.0 ± 10.8	67.5 ± 11.6	65.7 ± 10.7	0.316
Body height (cm)	161.4 ± 7.0	160.5 ± 6.3	161.5 ± 7.0	0.307
BMI (kg/m^2^)	25.3 ± 3.9	27.2 ± 3.2	25.0 ± 3.9	0.201
Preoperative D-dimer (μg/ml)	0.45(0.25,0.84)	0.91(0.61,1.12)	0.66(0.32,0.98)	0.045
APTT (s)	28.1 ± 3.8	28.1 ± 4.6	28.1 ± 3.7	0.968
PT (s)	12.0 ± 0.9	12.0 ± 0.8	12.0 ± 0.9	0.865
INR	1.04 ± 0.08	1.04 ± 0.07	1.04 ± 0.08	0.927
Platelet count (10^9^/L)	206.9 ± 61.7	194.8 ± 55.8	208.2 ± 62.2	0.193
Hb (g/L)	128.7 ± 18.4	126.1 ± 12.0	129.0 ± 18.9	0.091

*DVT* deep vein thrombosis, *BMI* body mass index, *APTT* activated partial thromboplastin time, *PT* prothrombin time, *INR* international normalized ratio, *Hb* hemoglobin

We divided patients aged > 50 into 10-year age groups. We found that the incidence of thrombosis in the group older than 80 years old was higher than that in the other groups, and the difference was statistically significant. In addition, we compared conventional D-dimer and age-adjusted D-dimer cut-off by age group; more patients below cutoff values could be found in all age groups using age-adjusted D-dimer cut-off values. The specific information is shown in Table 2.

**Table 2:** Comparison of different cut-off values stratified by age group

		Age range (years)			
	Total patients	50–59	60–69	70–79	> 80
No (%) of patients	406	103 (25.4)	182 (44.8)	99 (24.4)	22 (5.4)
Median age (years)	63.3	54.7	64.9	73.5	83.3
No (%) of patients with DVT	39	3 (2.9)	16 (8.8)	16 (16.2)	4 (18.2)
Conventional cut-off value					
No (%) of patients below cut-off value	226 (55.7)	73 (70.9)	99 (54.4)	46 (46.5)	8 (36.4)
Age-adjusted cut-off value					
No (%) of patients below cut-off value	269	78 (75.7)	118 (64.8)	6 0(60.6)	12 (54.5)

A total of 39 patients had found deep vein thrombosis of the lower extremities, all of which were asymptomatic. The incidence of thrombosis was 9.6%. Two of them were iliac vein thrombosis, and the rest were intramuscular venous thrombosis. The mean age of patients with thrombosis was 69.8 ± 6.9 years, and the mean age of patients without thrombosis was 65.0 ± 8.5 years, with a statistically significant difference (*p* < 0.001). For preoperative D-dimer levels, patients with thrombosis had an average of 0.91 (0.61, 1.12) mg/mL, compared with 0.66 (0.32, 0.98) mg/mL for patients without thrombosis, and there was a statistically significant difference (*p* = 0.045).

By multivariate logistic regression analysis, when age, gender, BMI, body weight, and preoperative D-dimer levels were independent variables, we found that only age and D-dimer levels were associated with the development of deep vein thrombosis (Table 3).

**Table 3:** Multiple logistic regression analysis for preoperative deep vein thrombosis

	OR	95% CI	*p* value
Age (year)	1.067	1.023–1.113	0.003
Sex (female)	0.615	0.208–1.814	0.378
Body height (cm)	0.977	0.910–1.049	0.52
Body weight (kg)	1.022	0.988–1.057	0.209
D-dimer (μg/ml)	1.331	1.032–1.716	0.027

*CI* confidence interval, *OR* odds ratio

**Fig. 1:**
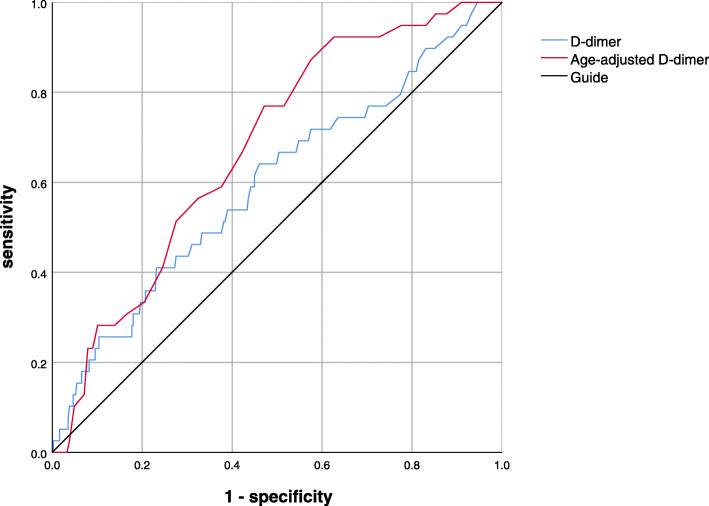
The ROC curve for the age-adjusted D-dimer and the traditional D-dimer

With regard to preoperative D-dimer, a total of 180 patients had a D-dimer concentration greater than 0.5 μg/mL, of which only 18 had thrombosis. With the use of age-adjusted D-dimer, there were only 33 patients’ D-dimer concentration above the age-adjusted D-dimer reference value. In addition, we plotted the ROC curve for the age-adjusted D-dimer and the traditional D-dimer (Fig. 1). The area under the curve was 0.795 and 0.685, respectively (Table 4). For DVT observations, the traditional D-dimer had a cut-off value of 0.845 with a sensitivity of 41% and a specificity of 0.658 (Table 4). The cut-off value of age-adjusted D-dimer cut-off value was 0.655, which caused a sensitivity of 0.769 and a specificity of 0.768 (Table 4).

**Table 4:** AUC and cut-off

	Cut-off value	Sensitivity	Specificity	AUC	95% CI	*p* value
Age-adjusted D-dimer	0.655	0.769	0.768	0.795	0.611–0.881	< 0.001
D-dimer	0.845	0.41	0.658	0.685	0.499–0.696	0.045

*AUC* area under the curve, *CI* confidence interval

## Discussion

Patients with joint replacement were generally older. If the traditional D-dimer value was used as a preoperative diagnosis method, the false positive patient would be greatly increased [5].

We used both age-adjusted D-dimer and traditional D-dimer values to detect the effectiveness of deep vein thrombosis. The area under the ROC curve was 0.795 and 0.685, respectively, and the age-adjusted D-dimer had a better predictive value in predicting deep vein thrombosis. In addition, we found that the sensitivity of the age-adjusted D-dimer cut-off value (76.8%) was significantly improved compared to the traditional method (41%), while the specificity, age-adjusted D-dimer cut-off value (76.9%) was also slightly better than the traditional D-dimer (65.8%).

The basic D-dimer value increased with age. Harper et al. found that D-dimer concentration increased with age reducing the clinical value of the D-dimer assay in the elderly [5].

Through the age-stratification study, we found that the elderly had higher incidence of thrombosis, which is consistent with the conclusion of our risk factor analysis, and by the age-adjusted D-dimer cut-off value in all age groups, it can be found that more samples are lower than the cut-off value, which improved the sensitivity of the model.

In our study, the age-adjusted D-dimer value was defined as age × 0.01 (μg/mL), which was considered safe. Nybo and Hvas had found that the age-adjusted D-dimer cut-off was safe to recommend the use of an age-adjusted D-dimer in a DVT setting as well as for PE from a systematic review [9]. In this study, we also made multivariate logistic regression analysis to find that age is the risk factor of DVT.

In previous reports, Imai et al. demonstrated that age and D-dimer index can be useful in screening patients for DVTs before THA [13]. The conclusion was similar to us. Moreover, age-adjusted D-dimer value was calculated as patients’ age times 0.01(μg/mL) by Schouten et al. [10]. They drew conclusions that age-adjusted D-dimer value can increase specificity, whereas sensitivity was not. The sensitivity was 97%, but the specificity was below 60%. The results did not coincide with our research. In addition, some studies focused on the performance of age-adjusted D-dimer cut-off values for reducing the use of ultrasounds [3, 14, 15]. Broen et al. inclined the view that age-adjusted D-dimer cut-off values can lead to less duplex ultrasounds performed to find patients that suffer from DVT [16].

In our study, the age-adjusted D-dimer value cut-off increased sensitivity, which was important to find patients with DVT. DVT was the most critical complication. Once we did not observe the asymptomatic DVT patients, it might cause serious consequences, such as pulmonary embolism (PE). Thus, the age-adjusted D-dimer could help us find more asymptomatic DVT patients. Actually, that was what we cared about more.

As far as we know, it was the first study to focus on the performance of age-adjusted D-dimer cut-off values for the screening of deep vein thrombosis in both TKA and THA. We gave the lower extremity venous color ultrasound bedside to all patients admitted to the hospital. Thus, we had enough samples for observation.

However, there were several limitations in our study. First, we did not use Wills score to assess the sensitivity and specificity of age-adjusted D-dimer cut-off value. Wills score could help us predict DVT better [17]. Second, our hospital used latex agglutination turbidimetry (LATEX) to measure the concentrations of D-dimer. Compared to enzyme-linked fluorescent assays (ELISA), LATEX may lead to lower sensitivity and specificity in our study. Finally, we did not group patients to several levels by age, which may get a better result in this study.

In conclusion, compared to traditional D-dimer, age-adjusted D-dimer showed better performance in screening DVT, which was useful clinically. Meanwhile, more clinical observations were needed to verify our ideas.

## Supplementary Information

**Additional file 1.** Supplementary Material.

## Data Availability

The data used to support the findings of this study are available from the corresponding author upon request.

## Abbreviations

DVT: Deep vein thrombosis
TKA: The knee arthroplasty
THA: The hip arthroplasty
TJA: The joint arthroplasty
PTE: Pulmonary thromboembolism
BMI: Body mass index
ROC: Receiver operating characteristic
AUC: Area under the curve
APTT: Activated partial thromboplastin time
PT: Prothrombin time
INR: International normalized ratio
Hb: Hemoglobin
CI: Confidence interval
OR: Odds ratio
PE: Pulmonary embolism
ELISA: Enzyme-linked fluorescent assays
LATEX: Latex agglutination turbidimetry
