# Supplementary material for: Performance of age-adjusted D-dimer values for predicting DVT before the knee and hip arthroplasty
Source: J Orthop Surg Res. 2021 Jan 25;16:82. doi: 10.1186/s13018-020-02172-w (PMC7831181; doi:10.1186/s13018-020-02172-w)
Supplement: Supplementary file 1 — Additional file 1. Supplementary Material. [file 13018_2020_2172_MOESM1_ESM.docx]

1. scatter plot of results


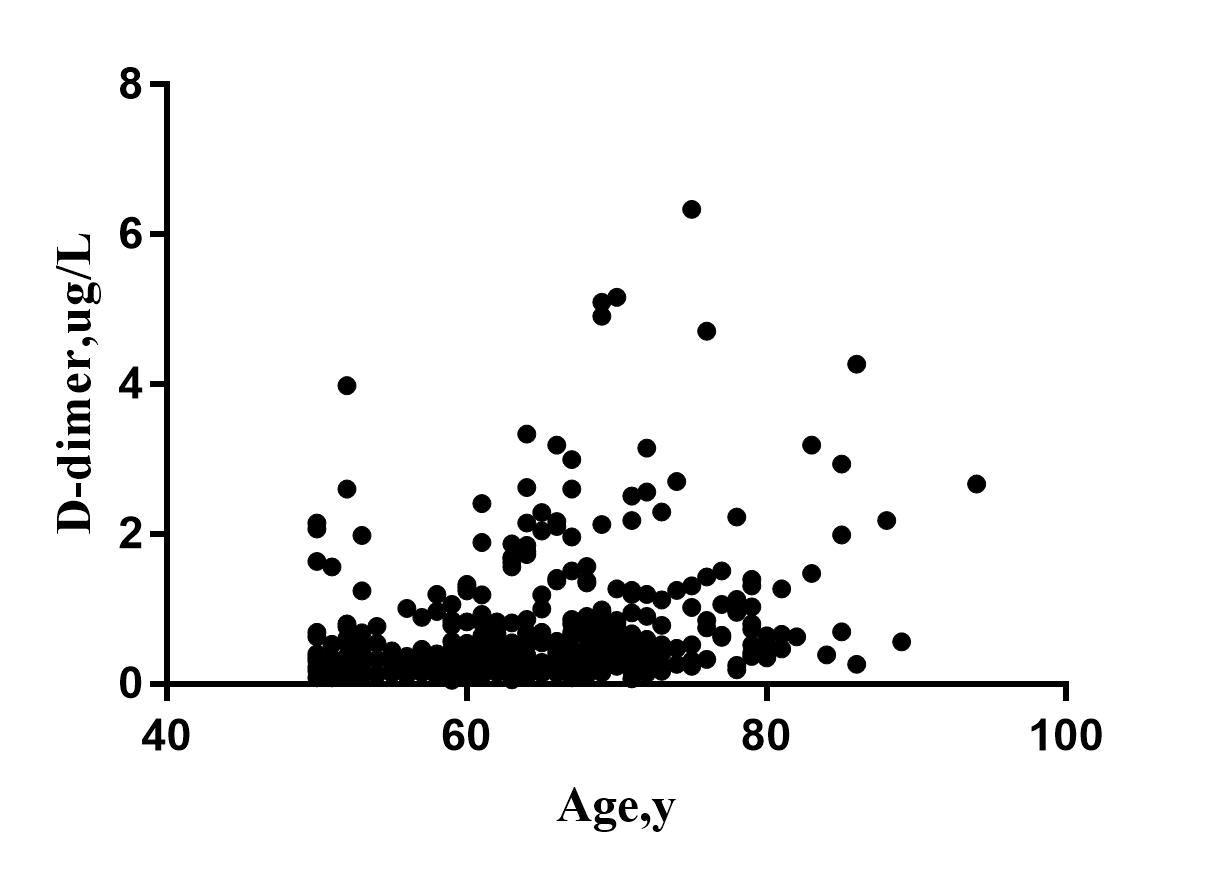


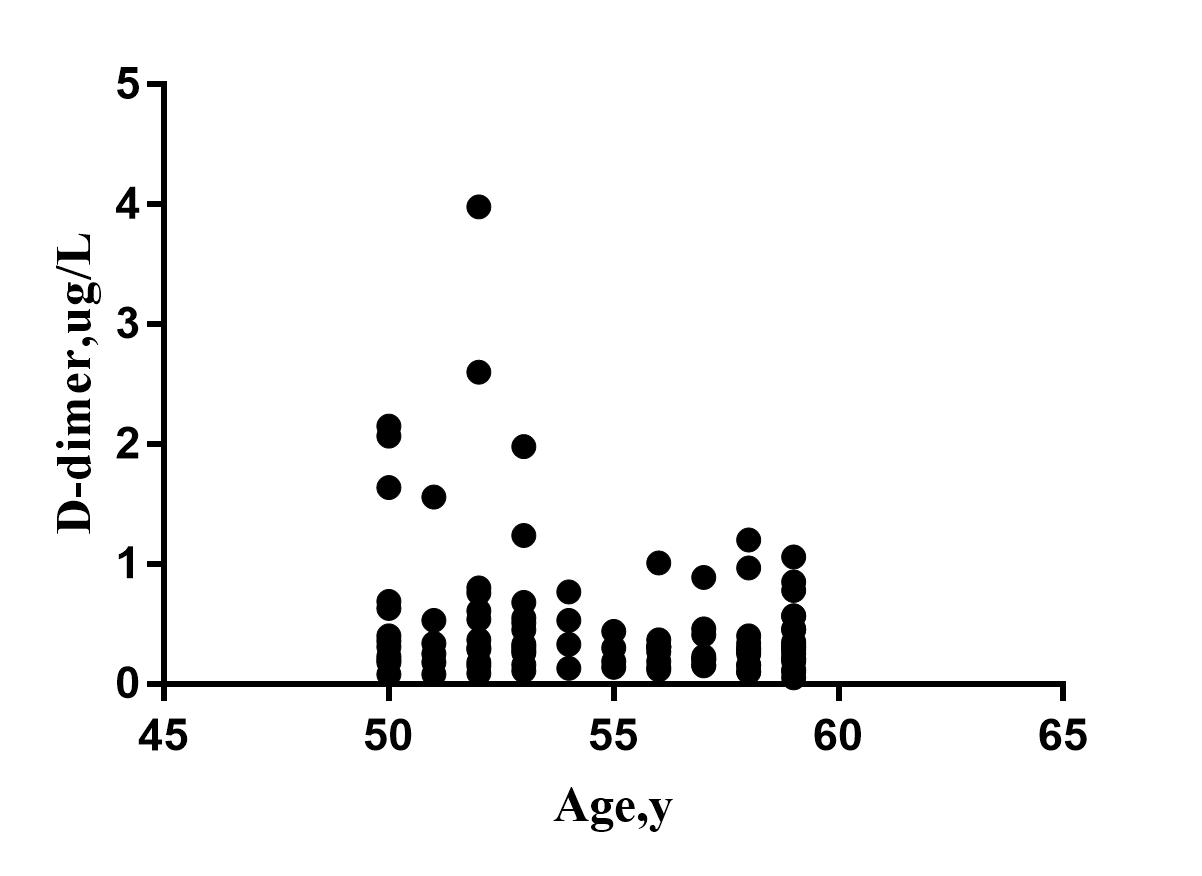


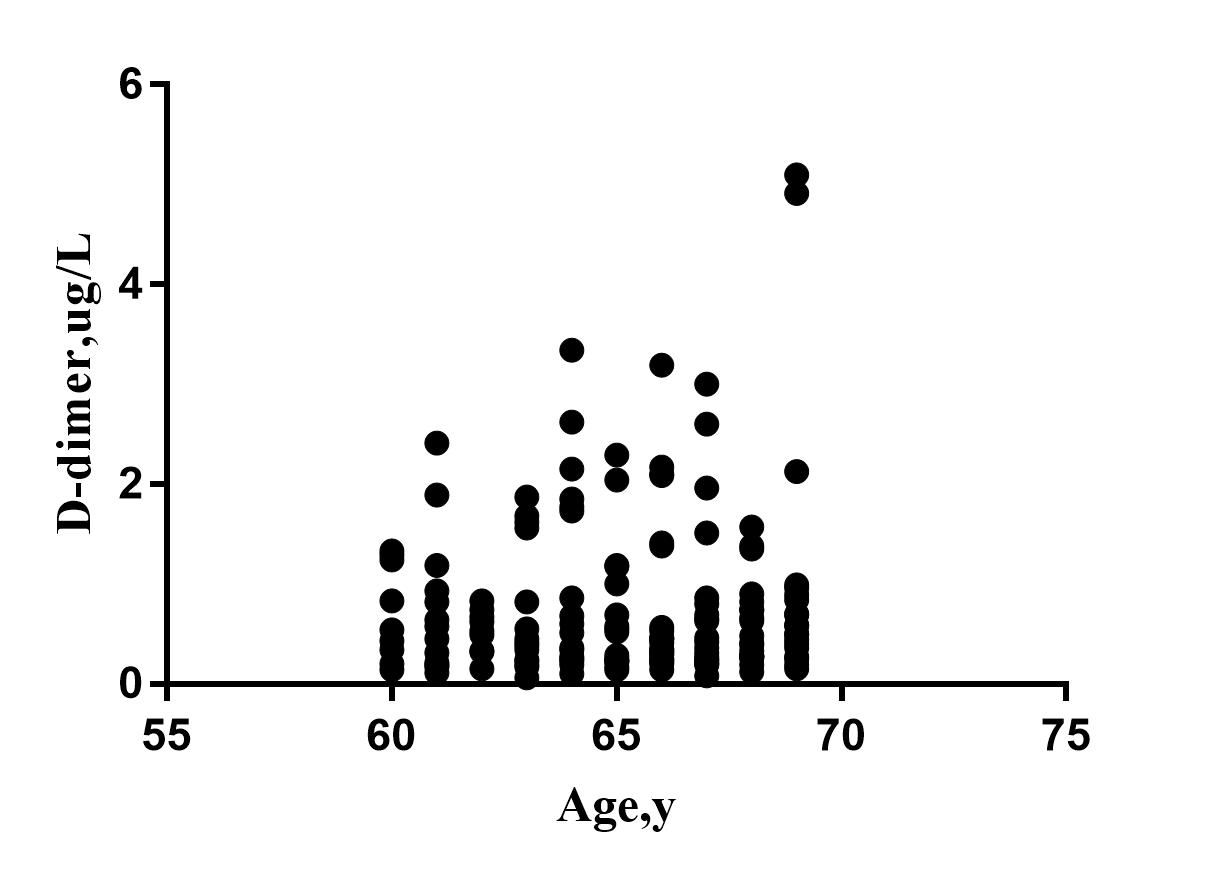


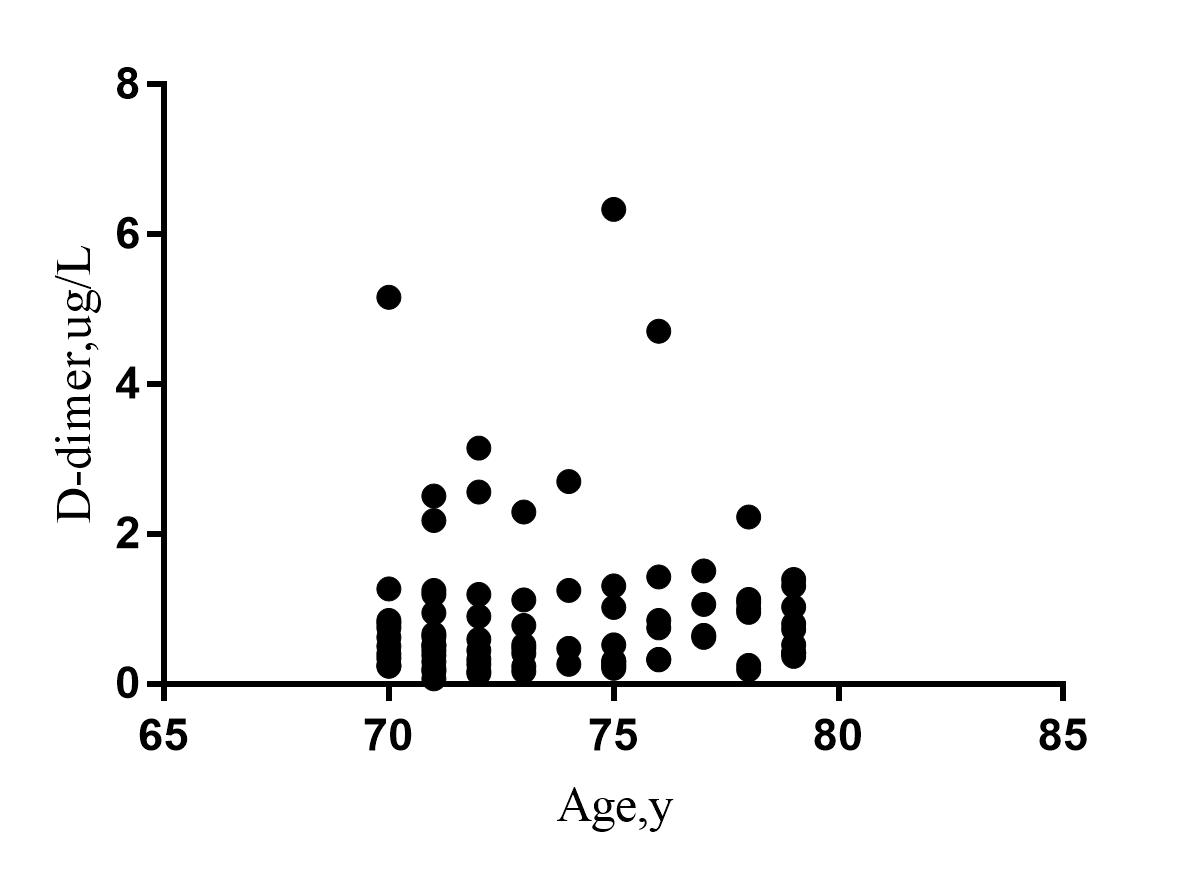


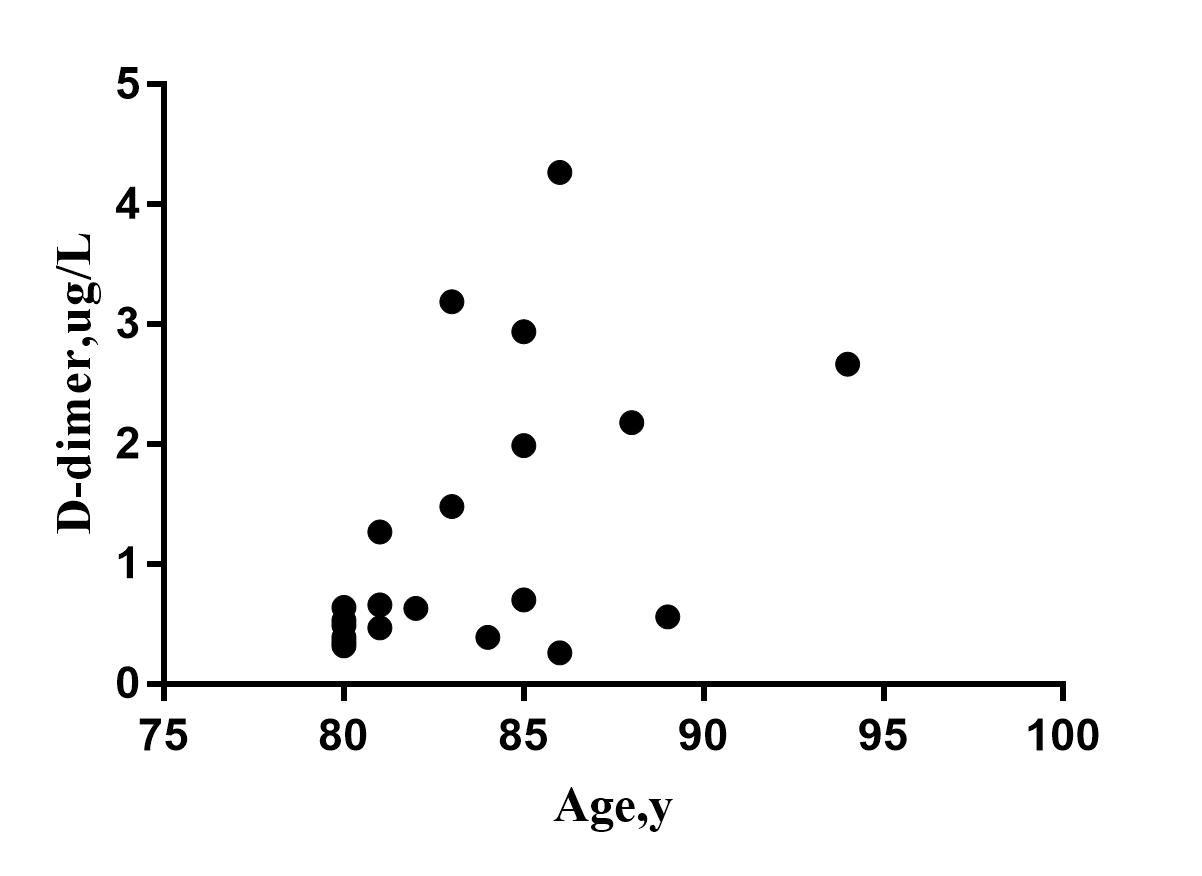


1. test of normal or skewed distribution

**1）combine**

**
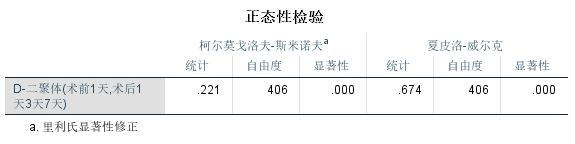
**

**
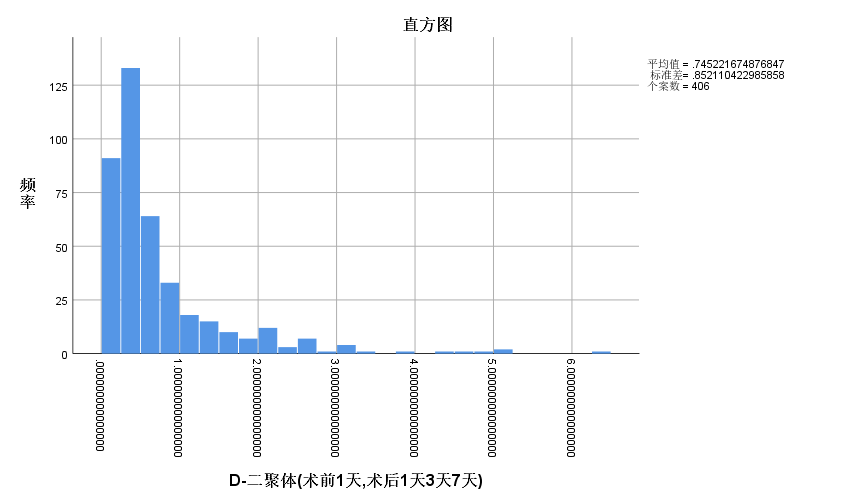
**

**2）Age 50-59**

**
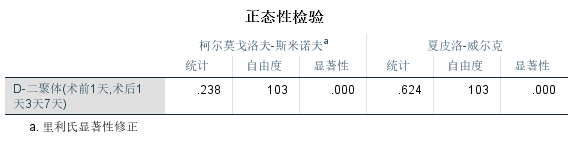
**

**
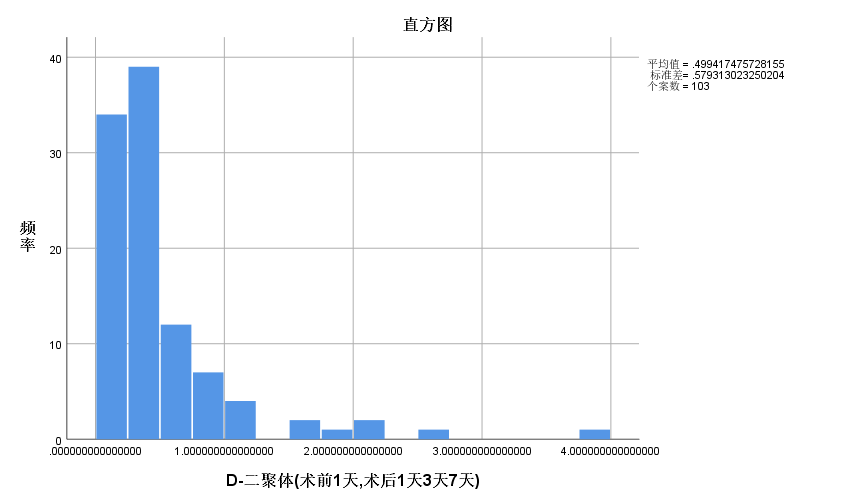
**

**the median 0.31**

**mean 0.49**

**SD values 0.57**

**3）Age 60-69**

**
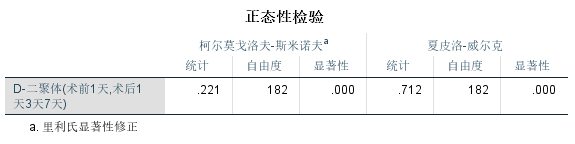
**

**
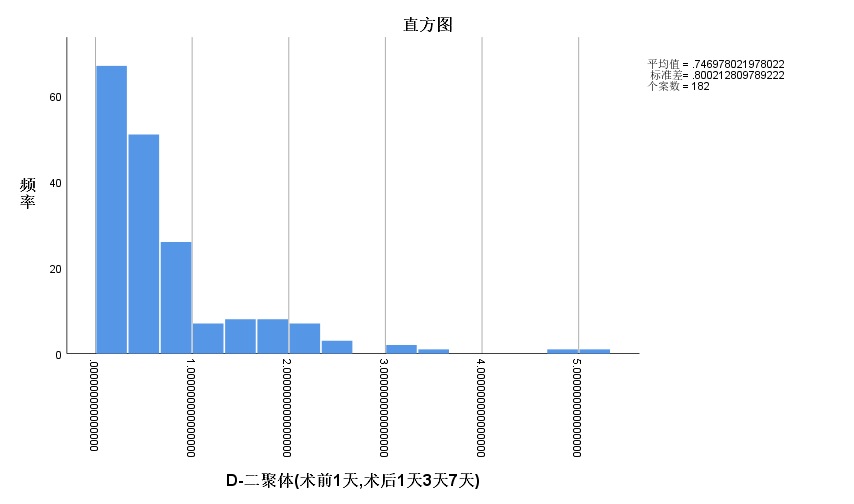
**

**the median 0.45**

**mean 0.75**

**SD values 0.72**

**4）Age 70-79**

**
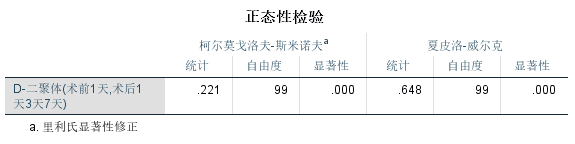
**

**
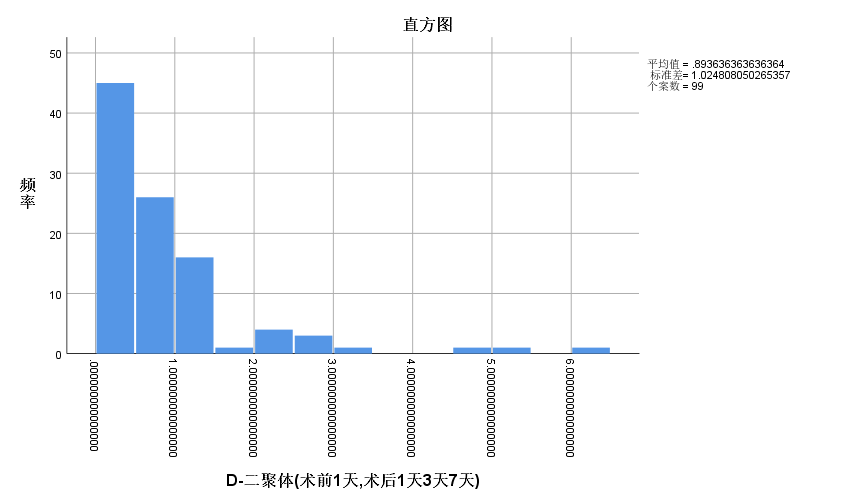
**

**the median 0.52**

**mean 0.89**

**SD values 0.87**

**5）Age >80**

**
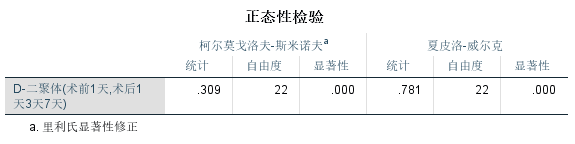
**

**
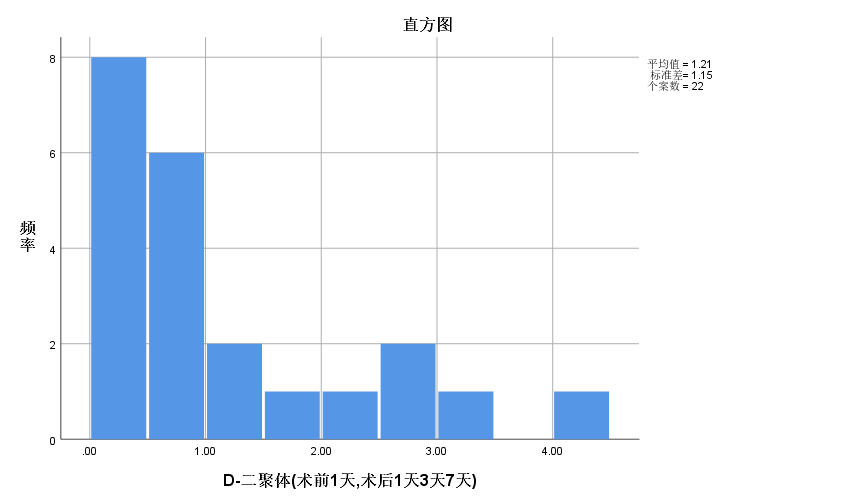
**

**the median 0.64**

**mean 1.2**

**SD values 1.1**

1. stratify your results based on increasing age-ranges

all 0.45(0.25,0.84)

50-59 0.31(0.19,0.54)

60-69 0.45(0.25,0.85)

70-79 0.52 (0.32,1.06)

>80 0.64(0.32,2.03）
